# How well can skin marker analysis detect the kinematics of a total ankle arthroplasty? - a comparison to videofluoroscopy

**DOI:** 10.1186/1757-1146-5-S1-O35

**Published:** 2012-04-10

**Authors:** Renate List, Hans Gerber, Mauro Foresti, Silvio Lorenzetti, Pascal Rippstein, Edgar Stüssi

**Affiliations:** 1 Institute for Biomechanics, ETH Zurich, 8093 Zurich, Switzerland; 2 Foot and Ankle Center, Schulthess Clinic, 8093 Zurich, Switzerland

## Background

Previous in vivo studies on total ankle arthroplasty (TAA) kinematics were mainly performed using skin marker analysis, which has the drawback of skin movement artefacts [1]. A further limitation is the inaccessibility of the talus for attaching markers, thus the impossibility to distinguish tibiotalar from subtalar motion. So far it is not known how well skin marker analysis detects the kinematics of the TAA.

## Materials and methods

The kinematics of 11 TAA participants were simultaneously analysed by skin marker and videofluoroscopic assessment during level gait (gt), walking up- (uph) and downhill (dnh). The fluoroscopic data analysis included a 2D/3D registration (error < 0.2° in-plane, <1.3° out-of-plane) [2]. The markerset consisted of 4 rearfoot and 6 shank markers [3]. For both approaches joint rotations were described along the axes of the marker based joint coordinate system. As a descriptor of differentiation the maximal and the root mean square differences (max diff, RMS diff) between skin marker and fluoroscopic joint rotations were calculated over the whole stance phase. Besides, maximal ranges of motion (ROM) were compared using a paired t-test.

## Results

Skin marker analysis significantly overestimated sagittal plane ROM of the TAA for 5(gt), 6(uph) and 6(dnh) and underestimated for 1(uph) and 2(dnh) subjects. Frontal plane ROM was significantly overestimated for 7(gt), 8(uph) and 9(dnh) of the 11 subjects. Transverse plane ROM was for 2(uph) and 2(dnh) subjects significantly overestimated, and for 3(gt), 1(uph) and 7(dnh) subjects significantly underestimated by skin markers. For mean RMS diff, mean max diff and mean ROM see Table 1.

**Table 1:** RMS diff and max diff over the whole stance phase and ROM assessed by videofluoroscopy (fluoro) and skin marker analysis (skin). Mean and SD over all 11 subjects of sagittal (sag), frontal (front) and transverse (trans) plane rotations, * statistically significant difference between fluoro and and skin (p<0.05).

		Level gait						Uphill						Downhill					
		Sag		Front		Trans		Sag		Front		Trans		Sag		Front		Trans	
**RMS diff [°]**	**Mean** **± SD**	1.5 ± 0.7		2.0 ± 0.9		2.9 ± 1.0		1.5 ± 0.6		2.4 ± 1.4		2.8 ± 1.2		1.7 ± 0.6		2.0 ± 0.9		3.0 ± 1.2	
**Max diff [°]**	**Mean** **± SD**	3.9 ± 1.7		4.9 ± 2.5		6.8 ± 2.7		3.6 ± 1.8		5.1 ± 2.8		5.7 ± 2.2		4.3 ± 1.7		4.5 ± 1.9		5.7 ± 1.9	

		**Fluoro**	**Skin**	**Fluoro**	**Skin**	**Fluoro**	**Skin**	**Fluoro**	**Skin**	**Fluoro**	**Skin**	**Fluoro**	**Skin**	**Fluoro**	**Skin**	**Fluoro**	**Skin**	**Fluoro**	**Skin**

**ROM [°]**	**Mean** **± SD**	10.0* ± 2.8	11.6* ± 2.9	2.9* ± 1.0	5.8* ± 2.1	8.1* ± 2.9	6.5* ± 2.7	9.6* ± 4.8	11.5* ± 3.3	2.5* ± 0.7	5.6* ± 2.7	6.6 ± 2.3	6.8 ± 3.2	13.1 ± 3.7	14.7 ± 2.7	3.0* ± 0.6	5.4* ± 2.1	7.7* ± 2.5	5.7* ± 2.3

## Conclusions

The differences between skin marker assessed rearfoot-shank and the fluoroscopic assessed isolated TAA motion were neither consistent between subjects, nor motion planes, nor conditions. For transverse and frontal plane rotations, the maximal differences were in the range of the maximal corresponding ROM. Discrepancies for the sagittal plane were smaller, but still for some subjects, ROM were significantly different.
